# A Place to Hide in the Home-Cage Decreases Yolk Androgen Levels and Offspring Emotional Reactivity in Japanese Quail

**DOI:** 10.1371/journal.pone.0023941

**Published:** 2011-09-29

**Authors:** Vanessa Guesdon, Aline Bertin, Cécilia Houdelier, Sophie Lumineau, Laureline Formanek, Kurt Kotrschal, Erich Möstl, Marie-Annick Richard-Yris

**Affiliations:** 1 UMR CNRS 6552, Ethologie Animale et Humaine, Université de Rennes 1, Rennes, France; 2 Konrad-Lorenz-Forschungsstelle, University of Vienna, Grünau, Austria; 3 Department of Natural Sciences, Biochemistry, University of Veterinary Medicine, Vienna, Austria; Pennsylvania State University, United States of America

## Abstract

An animal's emotional responses are the result of its cognitive appraisal of a situation. This appraisal is notably influenced by the possibility of an individual to exert control over an aversive event. Although the fact that environment controllability decreases emotional responses in animals is well established, far less is known about its potential trans-generational effects. As the levels of avian yolk hormones can vary according to the mother's environment, we hypothesized that housing environment of mothers would modulate the quality of her eggs and in turn her offspring's behaviour. Two groups of female Japanese quail were constituted: a group that had access to a place to hide in their home-cage (Hd, n = 20) and a group that had nowhere to hide (NoHd, n = 20) when stressed. Both groups were submitted to daily human disturbances for a twenty-day-period. Hd females produced eggs with both less testosterone and androstenedione than did NoHd females. The emotional and social reactivity of Hd females' offspring were lower and their growth was slower than those of NoHd females' offspring. Our results show that a minor difference in housing environment had substantial effects on eggs and offspring. The presence of a shelter probably helped quail to cope with daily human disturbances, producing less reactive offspring. This transgenerational effect caused by an opportunity to hide could lead to applications in care of laboratory animals, conservation biology and animal welfare.

## Introduction

An animal has to cope with various stressful situations in connection with foraging, predation and variations of its physical and social environment. An animal's emotional responses to these events seem to result from its appraisal of the situation. This appraisal is based on a limited number of elementary criteria such as novelty, intrinsic attractiveness, significance in relation to the individual's needs or expectations, and the possibility to control the situation [1]. The controllability criterion is defined as the capacity to produce responses enabling the individual to prevent, reduce or terminate an event [2]. Moreover, in many species, this controllability seems to be a fundamental element that affects their emotional responses to an aversive event. Rats able to press a bar to avoid an electric shock presented fewer physiological signs of stress, such as weight loss and gastric lesions, than those that could not exercise control, even though both groups were shocked and received exactly the same amount of shock [3]. The possibility for farm animals to exert control over their environment reduced the effects of aversive events [4] and decreased their fear reactions [5]–[6]. Aversive events before or during egg formation consistently influenced the quality of eggs and offspring phenotypes of several birds species [7], [8]. This suggests that the possibility to exert control over aversive events could moderate maternal effects.

Over the past decade, attention has focused on hormone-mediated maternal effects. Yolk androgen concentrations vary in response to the environmental conditions experienced by mothers of a wide range of species [8]. Various environmental factors experienced by laying birds such as, for example, breeding density [9], [10], frequency of social intrusions or social instability [11], [12], maternal social status [13], diet [14], unpredictable stressors [15] or male attractiveness [16] are thought to influence yolk steroid levels. Maternal yolk androgens can affect numerous offspring traits. For example, increase of yolk testosterone levels, in altricial birds, stimulates or impairs growth, increases overall activity, begging behaviour and frequencies of aggressive and sexual displays [7], [17]–[22]. Similarly, pre-hatch exposure to testosterone modifies social dominance among nest-mates [7] and modulates neophobic responses [23]. These phenotype changes are thought to adjust offspring's development to their post-hatching environment.

Despite a long history of domestication and selection, these offspring phenotype changes via maternally-derived yolk hormones appear well conserved in farm bird populations. Although, whether the behavioural impacts of androgens are adaptive or not is difficult to determine, these maternal effects are of considerable importance for the management of captive populations and must be investigated in more detail. Embryos of precocial species develop steroid-metabolising enzymes and steroid receptors very early during their development [24]. Yolk steroids seem to be metabolised, and then consumed by embryos during the first six days of incubation [25]. Impact of maternal androgens, especially yolk testosterone, on offspring behaviour and morpho-physiology has been evidenced by administrating physiological doses of hormones in eggs [26]–[30]. Quail yolk androgen levels vary with selection for behavioural traits, such as social motivation or fearfulness [31], [32]. To our knowledge very few studies have investigated which factors in laying females' environment are likely to affect offspring phenotype via yolk androgens. Previously we demonstrated that the quality of human-animal relationships as well as social instability during laying influenced yolk androgen levels. In turn, offspring growth, their emotional reactivity (i.e. propensity to express fear responses) and social motivation were found to be consistently influenced by these maternal environmental conditions [12], [29], [33].

The present experiment tested the hypothesis that the quality of maternal housing during laying could translate into maternal effects in quail. In the field, quail usually hide under a vegetal cover when they are disturbed [34], [35]. Thus, we hypothesized that providing a place for captive quail exposed to stressful events to hide would help them to cope with stressful events and would impact egg quality and offspring behaviour. To that end, two groups of female Japanese quail were maintained in two different types of home-cages and exposed to identical human disturbances. In one type of cage, the quail could hide behind a wall, whereas in the other type they could not. We analysed the quality of the eggs laid by the females and the behaviour of their offspring. As stressful conditions have been reported to increase androgen levels in Japanese quail as well as in other species [11], [15], [36], we predicted that quail that could not hide in their home-cage would produce eggs with higher levels of androgens and more fearful chicks than quail that had the possibility to hide when confronted with disturbances. We focused on yolk androgens (testosterone and androstenedione) because these hormones are the avian maternal hormones that have received the most interest so far and they have strong effects on embryonic development with long-lasting consequences [30]. Yolk progesterone levels were also assayed. Although progesterone is the main hormone surrounding avian embryos in the early stage of development [37], [38], it has received very little attention in the literature.

## Materials and Methods

Ethics statement

All our animal investigations were approved by the departmental direction of veterinary services (Ile et Vilaine, Permit number 005283) in accordance with the European Communities Council Directive of 24 November 1986 (86/609/EEC).

### Housing and treatment of laying quail

**Figure 1:**
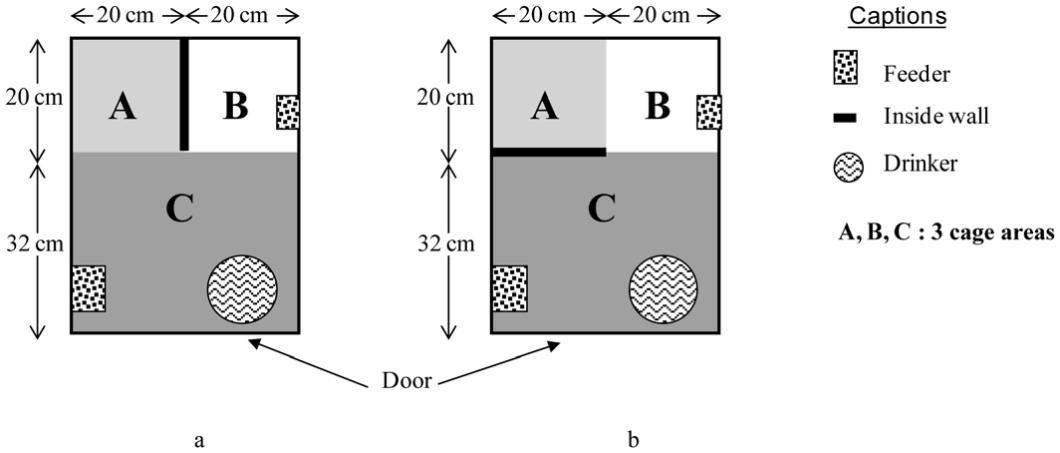
The design of home-cages with (Hd) or without (NoHd) the presence of a hiding place. (**a**) NoHd home-cage (*N* = 20) and (**b**) Hd home-cage (*N* = 20).

Forty two-month-old female Japanese quail from a commercial farm were used. Each bird was housed individually in a wire mesh home-cage (52 cm×40 cm×35 cm) with opaque lateral walls (they could hear but not see one another). All home-cages were equipped with similar drinkers and feeders as described in Figure 1. Each animal was tested in tonic immobility tests (see below) to estimate their emotional reactivity. Female quail were then allocated to two groups with tonic immobility scores and weights balanced between the two groups. The difference between the two groups was the disposition of an opaque wall (20 cm×35 cm) located either parallel to the home-cage door or perpendicular to the home-cage door (Fig. 1). Thus, one group had the possibility to hide behind the opaque wall during human disturbances (Hd females), whereas the other group could not (NoHd females). Twenty sexually mature males were kept individually in conventional cages (22 cm×20 cm×15 cm) with a feeder and a drinker. In order to avoid a male effect, a same male was allocated to an Hd female and a NoHd female to fertilise the eggs. These males were introduced for 20 minutes each day during the first three days and then every other day, into each female's home-cage. Food and water were available *ad libitum* during a 14∶10 h light∶dark cycle and the ambient temperature was maintained at approximately 21±2°C. All care was dispensed by the same person for the duration of the experiment.

### Human disturbances

Factors such as novelty, contact with humans, unpredictability, sudden or sharp movements are known to induce stress-effects in domestic fowl [39]. Therefore, to stress our subjects effectively, we combined the presence of a human being and sudden events subsequently called “human disturbances”. Following 40 days of acclimatisation to their individual home-cage, all females were exposed daily to identical human disturbances for a 20-day-period. Three kinds of disturbances were randomly applied during the light phase: 1) the experimenter rapidly opened the door of the home-cage, introduced a red flag, waved it for five seconds in the middle of the cage and then slammed the home-cage door; 2) the experimenter rapidly opened the home-cage door and rapidly introduced her right hand as far as the middle of the cage for three seconds and then slammed the home-cage door; 3) the experimenter noisily rubbed a wooden stick against all the doors of the home-cages, creating a sudden sound. These three actions were applied once a day at unpredictable times during the light phase. The experimenter changed her appearance by putting on a black poncho with a rain hood, a white sanitary mask, latex gloves and plastic boots before entering the rearing room to apply the disturbances so as to avoid habituating the subjects to the experimenter, as this experimenter also provided the animals' daily care. The location of each animal in its home-cage (A, B or C, Fig. 1) was recorded after disturbances 1) and 2).

### Sampling and measurements of eggs

Eggs laid before and during human disturbances were collected daily from each cage and weighed. The eggs laid from the ninth day of human disturbances until the end of these disturbances were stored at 16–19 C° as suggested by North [40] and were placed in a preheated, shelf-type, forced-draft incubator at 37.6°C and 45% humidity. All clear eggs were eliminated after one week of incubation. Two hundred and forty-one fertilised eggs remained: 120 from Hd females and 121 from NoHd females. The eggs were turned automatically twice a day. Three days before hatching (Day 14), rotation was stopped and the humidity was increased to 60%.

A group of 38 eggs (one per female) laid on the eighth day of human disturbances were kept frozen at −20°C until extraction for hormonal assays. Concentrations of yolk hormones were determined following the protocol described by Lipar *et al.* [37] and Hackl *et al.* [41]. The frozen yolk was separated from the albumin. Eggshells were separated, dried for 24 hours and weighed. The weight of the albumin was determined by subtracting the weight of the eggshell and the yolk from the weight of the whole egg. The concentrations of progesterone (P_4_) and androgens (androstenedione = A_4_ and testosterone = T) were assayed using a method similar to that described by Möstl *et al.* [38] and Hackl *et al.* [41]. As the distribution of hormones vary between egg layers [37], [41] the mixed yolk was assayed. Each yolk was suspended in 10 ml of water after being thawed and subsequently vortexed twice for 30 s in order to extract steroids. Samples were then stored overnight at 4°C. Afterwards, samples were vortexed and 1 ml of the suspension was transferred into a new vial. The suspension was then diluted with 4 ml of 80% methanol, vortexed for 30 minutes and stored at −20°C overnight to precipitate apolar lipids. After centrifugation (−15°C, 2500 g, 10 min) 10 µl of the supernatant were used for enzyme immunoassays [[for full descriptions of antibodies and validation see 38], [42], [43]. We measured yolk testosterone (T) in five assays, and androstenedione (A_4_) and progesterone in six assays. The inter-assay coefficients of variation were 19.5%, 10.4% and 15.9% for the low level pool and 12.3%, 5.5%, and 15.3% for the high level pool. The intra-assay variations were 8.5%, 4.2% and 9.2% respectively. The concentrations of hormones in the yolks are expressed in ng/g and the quantities of hormones in the yolks are expressed in g/yolk.

External calcification of the eggshell was measured in eggs laid before and during human disturbances (622 eggs before, and 561 eggs during the human disturbances). Higher external calcification of the eggshell is generally associated with higher levels of stress in poultry [44]. This extraneous calcification modifies the colour of the eggshell that can be assessed with a colour-measuring machine (Minolta CR2000) [44], [45]. Therefore, we estimated the effect of human disturbances on egg calcification by measuring the colour parameter “b” (yellowness).

Greater extraneous eggshell calcification means a greater difference between wet and dry eggshells and, consequently, a higher level of stress. The parameter “b” was measured on both poles of the eggs, once on the dried eggshell and once on the wet eggshell. The mean difference in “b” was calculated as follow: b_d_ = [(b_dry_-b_wet_)_small pole_+(b_dry_-b_wet_)_big pole_]/2. Then, before statistical analysis, we averaged the b_d_ of the eggs each quail had laid during the period before human disturbances and of the eggs laid during the period of human disturbances.

### Chick rearing

We obtained 58 chicks from females that could hide (Hd chicks) and 71 chicks from females that could not hide (NoHd chicks). After hatching, each chick was identified with a numbered ring on its leg. Sixteen sets of three chicks (each of these three chicks had a different mother) constituted each experimental group. The sex distribution was 23 females/25 males in the Hd group and 25 females/23 males in the NoHd group. The 32 sets of three chicks were all reared in the same room, in 32 wire-mesh cages (52 cm×40 cm×35 cm) with opaque lateral walls, a drinker and a feeder. Water and food (starter) were available *ad libitum* during a 14∶10 h light∶dark cycle. Ambient temperature was maintained at 37±1.5°C from hatching until chicks were eight-days-old and then, it was decreased progressively to 24±1°C, reached when they were 30-days-old.

All care was given by the same experimenter during all the experiment. The chicks were weighed at hatching, and when 14, 22, 28 and 35 days old.

### Behavioural characterisation of laying birds and chicks

Behavioural tests commonly used for poultry and described in the literature assessed the emotional reactivity and the social motivation of our subjects. Tonic immobility and hole-in-the-wall tests assessed emotional reactivity, whereas tests of separation from conspecifics assessed social motivation. The same person performed all the tests and wore similar clothing (i.e. white laboratory coat) at all times.

#### Tonic immobility test

This test followed a procedure similar to that described by Jones [46]. Animals were caught individually and carried to another room (about 2 m away from the rearing room). Each quail was then placed on its back in a U-shaped wooden cradle and held by the experimenter with one hand over the sternum and one gently covering the bird's head. The subject was restrained for 10 s prior to release. When more than 10 s passed between release and the bird's escape, duration of tonic immobility was measured. If not, another induction attempt was conducted. A score of zero second was given when tonic immobility could not be attained after five induction attempts. The test was stopped and a maximum duration of 310 s was allocated when the quail did not stand up within 310 s. The observer remained in the test room, but out of the quail's sight during the test. Duration of tonic immobility and number of induction attempts were recorded. All adult females before the stress period and at the end of the stress period, and all 14-days-old chicks were subjected to the tonic immobility test.

#### Hole-in-the-wall test

This test followed a protocol similar to that described by Jones *et al.* [47].The apparatus was a well-lit wooden box (62 cm×60 cm×33 cm) with a glazed observation window and had an opening for a hole-in-the-wall box on its perpendicular side. The floor was covered with wood shavings. A microphone was placed on top of the wooden box. Chicks were caught individually, carried in an opaque transport box (18 cm×18 cm×18 cm) and this transport box was placed in the hole-in-the-wall. Each chick was kept shut up in the hole-in-the-wall box for 1 min before the door of the hole-in-the-wall box was opened (habituation period). The experimenter recorded the number of exploratory pecks and the latency of the chick to emerge completely from the hole-in-the-wall box. Chicks failing to emerge within 3 minutes were discarded from the analysis (three chicks in each experimental group). Latency of first distress call and numbers of distress calls were extracted from the recordings. This test was carried out on chicks when they were 17–18 days old.

#### Separation tests

To assess the social motivation of our quail, each chick was separated from its two cage mates, once in its familiar environment, in its home-cage (12–13 days old), and once in an unfamiliar environment but in a cage similar to its home-cage (15-days old) following protocols adapted from Launay's and Launay *et al.*'s [48]–[49]. In the familiar situation, two chicks were taken and placed gently in an opaque transport box (18 cm×18 cm×18 cm) where they were kept until the end of test. The third chick was the test subject and remained alone in its home-cage for 3 min. A microphone was placed at the top of cage to record distress calls. As soon as the door was closed, the experimenter (out of the subject's sight) recorded the number of jumps. Latency of first distress call and numbers of distress calls were determined by analysing the sound recording. These chicks were also tested in an unfamiliar environment, in a cage similar to their home-cage. Each chick was taken out of its home-cage, carried gently in an opaque transport box (18 cm×18 cm×18 cm) for approximately 5 m and put in a cage similar to its home-cage for 3 min. A microphone was placed at the top of cage. The same parameters as in the familiar home-cage environment were recorded.

### Data analysis

Kolmogorov-Smirnov tests determined whether the data were normally distributed. None of behavioural tonic immobility tests, hole-in-the-wall-test and separation test variables were normally distributed. Therefore, they were analysed using non-parametric Mann-Whitney U-tests. We supplemented the behavioural analyses by Fisher's exact tests in order to test the proportions of chicks in each experimental group that required less than four inductions to induce tonic immobility, that emerged out of the box in less than 4 seconds and that jumped in both separation tests. Mann-Whitney U-tests analysed the frequencies (total of scans) of adult females located in areas A, B or C during human disturbances (total of disturbances 1 and 2). Hatching success and survival rates were tested using chi-square tests. Laying rates (number of egg per day per female) were analysed using a one-way ANOVA for repeated measures (experimental group×period). Log transformations of chick weight data, were analysed using a two-way ANOVA for repeated measures (experimental group×sex×age). The eggshell colour b_d_ parameter was analysed using a one-way ANOVA for repeated measures (experimental group×period). When required, we realised PLSD Fisher post-hoc tests.

The relative proportions of egg components (yolk and eggshell) were determined and analysed by multivariate analyses of variance (MANOVA). When Wilks' Lambda tests indicated a significant multivariate effect, individual one-way ANOVAs were performed for each dependent variable. Yolk hormone (T, A_4_ and progesterone) data were transformed by a logarithmic (X+1) transformation to meet the variance and covariance homogeneity requirements and analysed with a MANOVA and individual one-way ANOVAs. Data are presented as mean ± SEM. All analyses were performed using Statview software (SAS, Cary, NC), with significance accepted at *P*≤0.05.

## Results

### Behaviour of laying quail

Durations of tonic immobility and number of induction attempts did not differ significantly between the two groups of laying female quail either before or after human disturbances (before human disturbances: durations: 77.6 s±15.8 versus 75.5 s±15.4, Mann-Whitney *U*-test, *U* = 197, *P* = 0.94; number of inductions: 1.3±0.1 versus 1.3±0.1, Mann-Whitney *U*-test, *U* = 200, *P*>0.99; after human disturbances, durations 51.8 s±16.5 versus 51.3 s±7.9, Mann-Whitney, *U* = 140, *P* = 0.16; number of inductions: 2.2±0.3 versus 1.6±0.2, Mann-Whitney, *U* = 147.5, *P* = 0.18).

Hd quail were observed in area B (area close to the shelter for Hd group) of the home-cage during disturbances 1) and 2) significantly more frequently than were NoHd quail (0.21±0.02 versus 0.14±0.02, Mann-Whitney U-test, *U* = 113.5, *P* = 0.01). Frequencies of quail observed in areas A (shelter for Hd group) or C (front cage for both groups) did not differ significantly between Hd and NoHd quail (frequencies in area A: 0.19±0.03 versus 0.16±0.03, Mann-Whitney *U*-test, *U* = 182, *P* = 0.63; frequencies in area C: 0.61±0.04 versus 0.70±0.04, Mann-Whitney *U*-test, *U* = 138.5, *P* = 0.09).

### Egg laying and egg quality

Egg production did not differ significantly between Hd and NoHd females before, as well as during human disturbances (0.87±0.07 versus 0.89±0.05 egg/day and 0.86±0.03 versus 0.89±0.02 egg/day; ANOVA for repeated measures : group, *F_1,36_* = 0. 27, *P* = 0.61; period, *F_1,36_* = 0.02, *P* = 0.89; group×period, *F_1,36_* = <0.01, *P* = 0.99).

The b_d_ colour parameter did not differ significantly between eggs laid by Hd females and eggs laid by NoHd females either before human disturbances or during human disturbances (−3.0±0.5 versus −1.9±0.4 and −2.8±0.5 versus −2.7±0.5) (ANOVA for repeated measures : group, *F_1,35_* = 1.16, *P* = 0.29; period, *F_1,35_* = 2.42, *P* = 0.13). However, a significant group×period interaction emerged (*F_1,35_* = 7.86, *P*<0.01). The difference between wet and dry for b_d_ for NoHd quail eggs increased significantly between the period without human disturbances and the period with human disturbances, whereas b_d_ for Hd quail eggs did not differ between these two periods (PLSD Fisher, NoHd group, *P* = 0.01; Hd group, *P* = 0.34).

**Figure 2:**
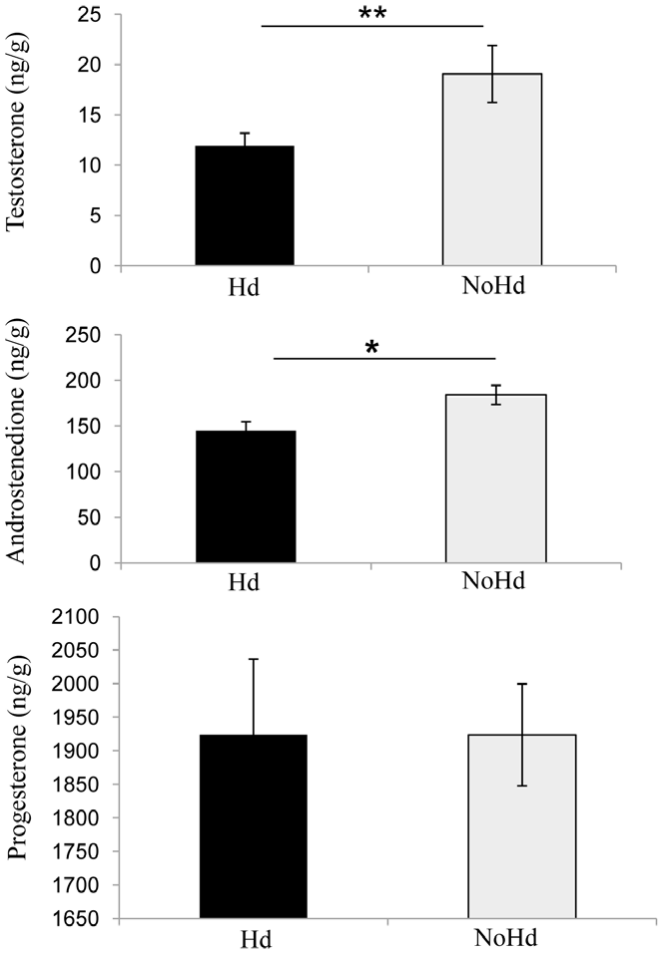
Yolk steroid levels in Hd nd NoHd females'chicks. Androstenedione (A4), testosterone (T) and progesterone (P4) concentrations (ng/g of yolk) in eggs of quail kept in cages either with (Hd group; *N* = 19) or without (NoHd group; *N* = 19) the presence of a hiding place. All data: means ± SE. Mann-Whitney U-test, * *P*<0.05; ** *P*<0.01.

We evidenced a significant overall effect of housing conditions on yolk hormone concentrations (Fig. 2) (MANOVA, *F*_3,34_ = 3.264, *P* = 0.03). Both T and A_4_ yolk concentrations were significantly lower in Hd females' eggs than in NoHd females' eggs, but P4 concentrations did not differ significantly between the two groups (T: *F_1,36_* = 5.31, *P* = 0.02; A_4_: *F_1,36_* = 7.22; *P* = 0.01; P4: *F_1,36_* = 0.00002, *P* = 0.99). Quantities of hormones per yolk differed significantly (MANOVA, *F_3,34_* = 63.37, *P*<0.01). Hd females' egg yolks contained significantly less of both T and A_4_ than did NoHd females' egg yolks, but quantities of P4 did not differ significantly. (T: 46.0±5.0 ng/yolk versus 76.6±11.8 ng/yolk, *F_1,36_* = 5.74, *P* = 0.02; A_4_ : 554.1±37.9 ng/yolk versus 742.8±52.5 ng/yolk, *F_1,36_* = 8.5, *P*<0.01; P4: 7380.0±434.9 ng/yolk versus 7789.9±456.6 ng/yolk, *F_1,36_* = 0.42, *P* = 0.52).

We found no significant overall effect of the treatment on the weights of egg components (eggshell, yolk and albumin; MANOVA, *F_3,34_* = 1.76, *P* = 0.17).

### Growth of offspring

Hatching success did not differ significantly between groups: 58 out of the 120 fertilised Hd females' eggs and 71 out of the 121 NoHd females' eggs hatched (Chi-2 test, *P* = 0.13). In addition to an obvious significant age effect (*F_4,368_* = 38659.15, *P*<0.001), chick growth tended to be affected by the treatment (*F_1,92_* = 3.56, *P* = 0.06), and a significant interaction between treatment and age emerged (*F_4,92_* = 3.64, *P* = 0.006). Growth of NoHd chicks appeared faster than that of Hd chicks during their first four weeks of life, although hatchlings' weights did not differ significantly (PLSD Fisher, *P*<0.05 from Day 14 to Day 28 and *P*>0.05 at hatch and D35; Table 1). Moreover, weight was influenced by chicks' sex (*F_1,92_* = 4.99, *P* = 0.03). Females were heavier than males. None of the interactions with the sex factor were significant (treatment×sex *F_1,92_* = 1.25, *P* = 0.27; age×sex *F_4,368_* = 1.51, *P* = 0.20; treatment×age×sex *F_4,368_* = 0.64, *P* = 0.64).

**Table 1:** Body weight (in g, means ± SE) at hatching and on days 14, 22, 28 and 35 post-hatching, of chicks born from eggs laid by quail kept in cages with (Hd, *N = 48*) or without (NoHd, *N = 48*) the possibility to hide from human disturbances.

	Body weight (g)	
Age	Hd chicks	NoHd chicks
Hatching	9.1±0.1	9.2±0.1
Day 14	76.8±1.5	82.0±1.4*
Day 22	142.4±2.1	149.5±2.3*
Day 28	185.7±2.5	192.8±2.5*
Day 35	223.5±3.1	227.8±2.9

Comparisons between the two groups for each age: PLSD Fisher.

*P<0.05.

### Offspring Behaviour

We could evidence no sex effect within each group for any of the behavioural data. Therefore, we pooled data for male and female chicks and did not include the sex factor in the behavioural analyses.

Tonic immobility tests revealed significant differences between the two groups of chicks. Significantly more induction attempts were required to induce immobility in Hd chicks than in NoHd chicks (Table 2). Moreover, proportionally significantly more Hd chicks required at least four inductions to induce tonic immobility than did NoHd chicks (17% versus 0%, Fisher's exact test, *P* = 0.006). However, immobility duration scores did not differ significantly between groups (Table 2). Hd chicks emerged significantly earlier from the box in hole-in-the-wall tests and expressed significantly more exploratory pecks than did NoHd chicks (Table 2). In addition, proportionally significantly more Hd chicks emerged from the box in less than 4 seconds than did NoHd chicks (74% versus 47%, Fisher's exact test, *P* = 0.03). Latencies of first distress call or numbers of distress calls did not differ significantly between the two groups (Mann-Whitney *U*-test, *P*>0.05 for all comparisons).

**Table 2:** Behavioural parameters (means ± SE) for the tonic immobility and hole-in the-wall tests for chicks born from eggs laid by quail kept in cages with (Hd, N _tonic immobility_ = *48* and N _Hole-in the-wall_ = *45*) or without (NoHd, N _tonic immobility_ = *48* and N _Hole-in the-wall_ = *45*) the possibility to hide from human disturbances.

Tests	Parameters measured	Hd chicks	NoHd chicks
**Tonic immobility**	Number of inductions	2.5±0.2	1.8±0.1**
	Duration (s)	55.9±4.7	67.2±7.7
**Hole-in the- hole**	Latency of full emergence (s)	10.0±3.2	11.7±2.7*
	Exploratory pecking behaviour	3.6±0.5	2.6±0.6**
	Latency of first distress call (s)	50.0±10.2	29.0±6.8
	Number of distress calls	53.9±7.4	61.3±7.2

Mann-Whitney U-test:

*P<0.05,

**P<0.01.

Separation tests revealed differences between the two groups. In the unfamiliar environment Hd chicks jumped significantly less (0.1±0.1 versus 0.3±0.1, Mann-Whitney *U*-test, *U* = 988, *P*<0.01), and emitted distress calls later (59.9 s±11.1 versus 17.6 s±5.4 Mann-Whitney *U*-test, *U* = 855, *P*<0.01) than did NoHd chicks. However, numbers of distress calls did not differ significantly between groups (Mann-Whitney *U*-test, *U* = 973, *P* = 0.19). In the familiar environment, Hd chicks jumped less than did NoHd chicks (0.15±0.01 versus 1.21±0.45, Mann-Whitney *U*-test, *U* = 833, *P*<0.01). Latencies of distress calling or number of distress calls did not differ significantly between groups (Mann-Whitney *U*-test, *P*>0.05 for all comparisons). Moreover, proportionally significantly less Hd chicks jumped during the two separation tests than did NoHd chicks (separation test in unfamiliar environment: 2% versus 17% and separation test in familiar environment 8% versus 35%, Fisher's exact tests, *P* = 0.03 and *P* = 0.003, respectively).

## Discussion

This study evidenced an effect of laying quail's housing conditions on yolk androgen levels and on offspring phenotype. As expected less androgens were found in eggs of females that had the possibility to hide from human disturbances, and the emotional reactivity of offspring exposed to these lower levels of androgens was lower.

The fact that Hd laying females were observed more frequently in area B (area close to the shelter for Hd group) of the cage than were NoHd females could indicate that quail felt reassured by the proximity of a place to hide. Saint-Dizier *et al.* [50] observed that quail spent more time near a place to hide when exposed to a stressful situation (novel object) than in a situation without stress. In addition, even though tonic immobility tests did not reveal differences in fearfulness between NoHd and Hd laying females, eggshell calcification differences suggested that Hd females and NoHd females perceived the repeated human disturbances differently. Indeed, the external shell calcification of eggs laid by females having no place to hide during human disturbances was more important than that of eggs laid by females having a place to hide. A similar effect on external calcification of eggshells was observed previously in poultry, and high calcification levels are generally associated with high levels of stress [44]. This result suggests that Hd females probably perceived human disturbances as less stressful than did NoHd females.

Eggs laid by Hd quail contained less androstenedione and less testosterone of maternal origin than did eggs laid by NoHd quail. This was true for concentration levels as well as for the total quantities of each androgen per yolk. In addition to external eggshell calcification, this result suggests that Hd and NoHd quail probably perceived human disturbances differently in relation to their ability to exert a control over the situation. As suggested by Saint-Dizier *et al.* [50], the proximity of a protective cover could help birds to cope with stressful events and may moderate the impact of aversive events.

The modulation of farm birds' yolk hormone levels by environmental stimulation has only very recently been highlighted. So far, very few environmental factors have been identified as potential sources of inter-female differences in yolk hormone levels. Previously, we evidenced a strong influence of human-animal relationships or social environment on yolk androgens levels [12], [32]. Mild unpredictable stressors increase quail testosterone yolk concentrations [15] whereas long-term restraint stress lower testosterone yolk concentrations [51]. Housing conditions (floor-housed or caged hens) modulate domestic hens' yolk androstenedione and estradiol concentrations [52]. Hens' yolk estradiol levels are also modulated by chronic unpredictable food access [53]. We reported here, for the first time, an impact the layout of home cages on yolk steroid levels and, more particularly, an impact of the possibility to hide from disturbances. Like in altricial species, an interesting discrepancy was observed concerning the direction of variation of yolk hormone levels. Our results, agreeing with Guibert *et al.*'s [12], [15], indicated that quail yolk androgens increased after a stress, whereas other authors reported a decrease after exposure to stressful events [33], [51]. The nature, intensity, duration of exposure to aversive events or animals' age can all affect differently the physiology of laying birds and the quantities of hormones (and other compounds) transferred to the yolk.

According to our expectation, environmental challenges during laying have consequences on offspring phenotype. Mothers' possibility to hide in their home-cage significantly influenced growth of their offspring. Weight gain was higher in NoHd chicks than in Hd chicks during their first weeks after hatching, whereas no differences in weight were observed between groups at hatching. Therefore, NoHd chicks' growth rates were higher during early post-hatching development. Similarly, previous reports showed higher weight gains of young Japanese quail or Bobwhite quail chicks exposed to higher levels of prenatal androgens [28], [32]. Moreover, similar findings have been reported concerning other avian orders (canaries [7]; gulls [18], [20]; blue birds [54]; starlings [21], but see [26], [55]). This result could be explained by foraging or metabolic rate differences, but these parameters were not measured in the current study.

In addition, as expected, the emotional and social reactivity of Hd chicks was lower than that of NoHd chicks. Indeed, the fact that Hd chicks required more inductions in tonic immobility tests before remaining immobile indicates lower sensitivity to this test [47], [56] and thus lower general underlying fearfulness. Furthermore, the fact that Hd chicks emerged sooner in hole-in-the-box tests and explored more also indicates lower emotional reactivity [57], [58]. Jumps against the wall in separation tests are commonly interpreted as attempts to rejoin conspecifics [59], [60]. Hd chicks jumped systematically less than did NoHd chicks in separation tests, thus indicating their lower social motivation. Hd chicks developed a more “proactive” behavioural profile (sensus Koolhass *et al.* [61]) than did NoHd chicks despite their pre-hatch exposure to lower androgen levels. These data are in contradiction with other reports. Indeed, quail chicks from testosterone-injected eggs were previously reported to be less fearful, less socially dependant and to approach novel objects more rapidly than did chicks from non-injected eggs [27]; in other words these chicks exposed to higher testosterone levels before hatching developed a more “proactive” behavioural phenotype than did control chicks. On the other hand, another study evidenced that higher levels of androgens were associated with higher fearfulness and lower social motivation levels in quail chicks [33]. In addition, quail chicks exposed to higher testosterone concentrations were more fearful [12], [29], [62]. However, selection of more fearful birds was associated with less androgen [33]. Discrepancies between these studies and our results could be due to the implication of hormones other than testosterone in the development of emotional states (e.g. androstenedione and/or progesterone). In addition, other factors, like overall egg quality or a dose-dependent effect could explain these contradictions concerning the effects of androgens on quail chicks' behaviour. Indeed, hormone dose-response curves are often non-monotonic, but present an inverted U-shape, with intermediate doses having greater effects than either higher or lower doses [63]. Nevertheless, these contradictory results indicate that the direction in which yolk androgens can influence the course of development is not unique and can be somewhat unpredictable. Contradictory results have also been reported concerning altricial species [64].

Another important point is that other compounds or hormones could have contributed to the observed differences. Currently, growing evidence indicates that exposure of avian embryos to elevated corticosterone levels affects their morpho-physiology and behaviour after hatching [65]. It could thus be thought that the treatment had affected circulating corticosterone levels differently in relation to housing conditions and elicited correlated changes in yolk corticosterone concentrations. We still know little about the transfer of plasma corticosterone to the egg yolk. As mentioned by Henriksen et al. [65], one consistent result of studies of avian embryos exposed to elevated corticosterone levels is lower body mass or growth rates after hatching. NoHd females may have perceived human disturbances as more frightening due to their impossibility to hide behind a wall. Higher plasma corticosterone levels and correlated changes in yolk corticosterone could be expected. However, the fact that the mass gain of NoHd females' chicks was higher does not support the hypothesis of their greater exposure to stress hormones. This transfer was observed mainly by using non-physiological levels of circulation corticosterone (implants: [66]; feeding: [67]) and these high concentrations only induced a minor transfer [67]. Although some authors report a transfer of corticosterone to eggs and natural variations within- and between-clutches (e.g. European starlings, Sturnus vulgaris, [68]–[69]; barn swallows, Hirundo rustica [70]), other authors do not. Administration of ACTH increases adrenocortical activity, but does not influence corticosterone concentrations in hens' yolk [67]. Janczak et al. [71] and Nätt et al. [53] reported an influence of pre-hatch stress on offspring behaviour without any effects on egg corticosterone concentrations. Although this hypothesis cannot be totally rejected, we argue, following Rettenbacher et al. [67] and Janczack et al. [71], that the effects of maternal stress were probably mainly mediated indirectly. Furthermore, mild chronic stress, including human disturbances, did not affect Japanese quail plasma corticosterone levels [72]. In addition, the presence of high concentrations of various gestagens and gestagen metabolites (structurally similar to corticosterone) in quail egg yolks could also produce immunoreactive “corticosterone” as the concentrations of corticosterone in yolks are low compared to, for example, progesterone [73]. The presence of corticosterone remains controversial and, as suggested by Rettenbacher et al. [67], HPLC separations should be used to characterise these immunoreactive substances.

The known organisational and activating effects of hormones during early development provide a potent pathway by which mothers can modify their offspring's development [74]. The results of our present study contribute to the growing appreciation of non-genetic maternal influences on offspring phenotype in birds. Indeed, our data indicate that laying females' living conditions have transgenerational effects on offspring growth and behaviour, and that laying females' perception of their living context influences their offspring's development. Although behaviour of Hd females differed insignificantly from that of NoHd females, the possibility for Hd birds to exert control over aversive events affected significantly the quality of their eggs and their offspring's development. Thus, non-genetic maternal influence in birds is a very sensitive mechanism that reflects the living conditions of a mother and the way she perceives them. Moreover, our data could open new perspectives in the animal welfare domain. Indeed, one way to improve animal welfare for domestic species is enrichment of rearing environments. Environmental enrichment increases animals' ability to cope with challenges, enhances their behavioural repertoire, increases positive use of their environment and reduces or eliminates stereotypic behaviour [75]–[77]. Our results show that the assessment of the effectiveness of enrichment procedures (via increase of the ability to control the environment) should not be limited to the animals to which the procedures are applied, but should also be investigated on a transgenerational scale.
